# Clinical analysis of multiple flaps in repairing pressure injuries

**DOI:** 10.3389/fsurg.2025.1672663

**Published:** 2025-11-11

**Authors:** Huwen Wu, Hanbin Deng, Jiangling Yao, Jian Yang, Rong Wang, Peishen Zhang, Han Zhou, Shaowen Cheng

**Affiliations:** Department of Wound Repair, Key Laboratory of Emergency and Trauma of Ministry of Education, Key Laboratory of Hainan Trauma and Disaster Rescue, The First Affiliated Hospital, Hainan Medical University, Haikou, China

**Keywords:** pressure injury, flap, wound, repair, regeneration

## Abstract

**Background:**

Pressure injuries exhibit high prevalence and are cost-consuming in clinical treatment, and flaps are the most appropriate way for their repair, especially stage III and IV. This study was conducted to investigate the repair effects of different types of flaps and their precautions.

**Methods:**

This study retrospectively analyzed the clinical data of 33 patients with stage III and IV pressure injuries who were hospitalized in our department from April 2021 to December 2023. Data subjected to analysis included the location and size of pressure injuries, treatment, postoperative flap survival, complications, and wound healing after 6 months of follow-up.

**Results:**

Of the 33 patients involved (1 case in stage III and 32 in stage IV), there were 23 cases of sacral pressure injuries, 5 cases of ischial area, 3 cases of hip, 2 cases of back, 19 cases of fascia flaps, 5 cases of musculocutaneous flaps, 9 cases of perforator flaps, 31 cases of postoperative flaps survival, as well as 2 cases of wound healing after debridement and dressing change or re-transplantation of flaps. Furthermore, 4 cases developed complications such as incisal margin necrosis, fat liquefaction, and subcutaneous hematoma, which healed after further debridement and dressing change.

**Conclusion:**

Pressure injuries of different locations, stages, and sizes should be repaired individually with different types of flaps, with emphasis required for perioperative management, as well as postoperative nursing and treatment.

## 1 Introduction

Pressure injuries refer to long-term pressure and/or shear force-induced local damage of the skin and deep soft tissues. Such type of injuries are common in patients who are bedridden or in wheelchairs for a long time, such as those with paraplegia, coma, vegetative patients, Alzheimer's disease, spinal cord injuries, or related to medical devices (1). Currently, pressure injuries are highly prevalent in the clinical setting. In the United States, 2.5 million patients are treated for pressure injuries annually (2); in the Netherlands, the prevalence of pressure injuries is close to 13% in university-affiliated hospitals, 23% in general hospitals, 30% in nursing homes, and 12% in home care; in the UK, the rate is 21.8%, and Canada has similar results; in China, the prevalence rate in 12 general hospitals reaches 1.58%, and 13.47%–14.58% of which are cases in stage III and IV requiring surgical intervention. Pressure injuries also impose a heavy burden on the social economy. For example, the treatment cost of pressure injuries is about $11 billion in the United States, while the annual treatment cost is about 1.8–3.2 million pounds in the UK (3, 4). In addition, pressure injuries also bring a heavy psychological and life burden to patients and their families.

According to the Clinical Practice Guidelines for the Prevention and Treatment of Pressure Ulcers/Injuries (2019 edition), pressure injuries are mainly divided into six stages (5). Specifically, stage I–II can be treated conservatively, while stage III–IV, given their location in the deep tissue and even bone, require surgical treatment to provide the most direct and effective management (6, 7). At present, pressure injuries are generally treated by the following approaches, including: (1) Wound dressing: Foam dressing works to cushion pressure and absorb exudate; silver alginate ion dressing can also be antibacterial; debridement glue (known as hydrogel dressing) is generally applied for pressure injuries at or below II stage to facilitate wound autolysis, debridement and wet healing (8). (2) Debridement combined with ozone water: This method can keep a relatively clean wound to promote the growth of fresh granulation tissue through repeated debridement and washing with ozone water; (3) Vacuum sealing drainage (VSD): VSD can enhance a continuous drainage of wound exudate and inflammatory medium, and reduce bacterial colonization. In addition, the proposed wound therapy under continuous negative pressure can improve the blood supply of the wound, reduce tissue edema, and promote granulation tissue formation (9). (4) Platelet-rich plasma (PRP) and growth factors: With abundant growth factors and cytokines of various types, PRP can promote the formation of extracellular matrix during ound healing (10). In addition, PRP and biological products rich in growth factors are usually applied in combination with VSD to promote wound healing (11). (5) Skin grafting: It is frequently employed for treating relatively clean wounds after surgical debridement. It is simple to operate and can close the wound as soon as possible, which is therefore more suitable for application in primary hospitals (7). In general, pressure injuries, characterized by complicated wound surface and severe infection after bacterial invasion, cannot be managed effectively through local dressing change, and other non-operative methods, which are time-consuming in treatment and has a high probability of recurrence. Moreover, for some pressure injuries of buttocks, greater tension may be triggered owing to steps such as direct pulling and suturing after debridement, posing great challenge for the wound to heal. In addition, the skin graft is not wear-resistant after grafting, resulting in higher risk of recurrence and infection. Therefore, various types of flaps stand out for their applicability in the management of some III and IV pressure injuries (12).

In most cases, patients with pressure injuries are the elderly who are bedridden for a long time and have various underlying diseases concurrently. In nature, pressure injuries are chronic consumptive disease complicated with serious infection, and patients may often experience several comorbidities such as hypoproteinemia and anemia, highlighting the significance of preoperative evaluation and perioperative management. Accordingly, the present study was conducted to retrospectively analyze the demographic characteristics, systemic conditions, and wound location of eligible patients according to the pre-set inclusion and exclusion criteria, with the formulation of an individualized treatment plan. This study is anticipated to provide potential references for determining different flaps in clinical practice, and underlines the importance of perioperative comprehensive treatment in the treatment of patients with pressure injuries.

## 2 Materials and methods

### 2.1 Research design

This retrospective study was conducted in the Department of Wound Repair, the First Affiliated Hospital of Hainan Medical University from April 2021 to December 2023. Inclusion criteria: (1) Patients with stage III–IV pressure injuries according to the National Pressure Ulcer Advisory Panel (NPUAP) in the United States; and (2) patients (and their family members) who were informed of the surgical risks and provided written surgical informed consent. Exclusion criteria: (1) Patients who could not tolerate surgery owing to the presence of serious disorders involving the heart, lung, brain and other parts; (2) patients without follow-up data or with incomplete case data; (3) patients with uncontrolled infection of the wound; (4) patients with post-adjustment hemoglobin level of <90 g/L, scarce blood type or failed to obtain preoperative blood preparation; (5) patients with malignant tumors; (6) patients who could not cooperate due to mental illness; and (7) patients who were not suitable for flap transplantation for other reasons determined by the surgeon.

### 2.2 Treatment methods

#### 2.2.1 General treatment

Considering the high-risk population, i.e., the elderly with various underlying diseases and poor cardiopulmonary function, systemic nutritional support should be given in these patients after admission to improve the basic physical condition, control blood glucose and blood pressure, and avoid cardiovascular and cerebrovascular accidents. Supplemental albumin therapy should be applied for patients with poor nutritional status or hypoproteinemia. As reported previously, the daily protein consumption should be 1.5–3.0 g/kg to better promote wound healing (2). Transfusion therapy should be adopted for patients with low hemoglobin and blood transfusion indication. All patients had electrolyte balance and stable biochemical indexes during the perioperative period. Meanwhile, the family members or companions of patients were instructed to avoid further pressure on the local wound. A preoperative MRI examination of the affected site was performed to evaluate the soft tissue damage. With the utilization of general bacterial and fungal culture of wound secretions, sensitive antibiotics were used for anti-infection treatment. Finally, operation for wound debridement was scheduled for patients who could tolerate surgery and anesthesia, as evidenced by stable general condition.

#### 2.2.2 Wound debridement and VSD

Different modalities for anesthesia were selected according to different parts of pressure injuries. Necrotic tissue and fascia should be cleaned intraoperatively. If necessary, the wound should be extended and the dead space should be opened until the seepage of fresh blood on the tissue surface. After complete hemostasis, the wound was washed with 3% hydrogen peroxide, a large amount of normal saline, and diluted iodophor. PU or PVA materials used for VSD were trimmed depending on the size and depth of the wound, with the wound fully covered by film. The negative pressure of VSD and the smooth drainage tube were maintained after surgery, with support therapy continued postoperatively. The suction pressure of VSD is maintained at 125 mmHg–450 mmHg. The wound was opened 5–7 days after surgery. Debridement, and VSD would be continued in case of necrotic tissues on the wound. In addition, a second phase of flap repair treatment would be scheduled in the presence of fresh wound granulation tissues and no obvious necrotic infected tissues.

#### 2.2.3 Flap wound repair

A personalized treatment plan was established according to different affected sites and the systemic condition of patients. Clinically, flaps (e.g., fascial flap, musculocutaneous flap, and perforator flap) are common choices for repairing stage III and IV pressure injuries with deep wound surface, through rotation, propulsion, translocation, and propeller, with better effect as well.

A series of preparations are required before flap transfer, which are summarized as follows.

##### 2.2.3.1 Flap design

According to the location, area, and depth of pressure injuries, the flap was individually designed and marked with a sterile marker. Noticeably, perforator blood vessels would be detected and positioned with Doppler ultrasound preoperatively, with corresponding marks made, when utilizing the perforator flap.

##### 2.2.3.2 Methylene blue labeling and thorough debridement

The necrotic and deactivated tissues as well as aging granulation tissue of the wound were labeled with methylene blue solution and stained for subsequent complete removal. Then, the wound was washed with a large amount of normal saline and diluted iodophor after thorough hemostasis.

##### 2.2.3.3 Incision and transfer of the flap

A liquid with 1 mg/ml epinephrine added into 500 ml of normal saline was extracted with a 5 ml syringe and injected into the subcutaneous tissue along the designed marking line of the flap. After injection, the skin was swollen along the marking line, which could effectively prevent bleeding at the skin margin during flap incision and maintain a clear surgical field. Dissect the flap layer by layer along the flap marking line, and then rotation, propulsion, and translocation to repair the wound. The skin and subcutaneous tissues should be sutured discontinuously by a horizontal mattress inversion suture. Rubber strips can be placed intermittently on the closed wound for drainage. Following suture, the incisal margin should be covered with sterile dressing and the center area of the flap should be exposed to facilitate observation of flap survival. Alternatively, the opposite closure can be covered with a VSD device again for full drainage and complication prevention after the flap repair.

#### 2.2.4 Postoperative management

Patients were provided with general treatment after surgery continuously, including monitoring the basic physical condition, active treatment of the underlying diseases, correction of the acid-base balance and electrolyte disorders, nutritional supplementation, high-quality protein supplementation, wound healing, systemic antibiotics to prevent infection, and basic anticoagulant treatment. Simultaneously, it was essential to adopt careful postoperative care for patients, including avoiding long-term compression of the same part to trigger new damage, compression of the flap to reduce blood supply to the flap and affect the survival of the flap, and greater tension of the flap. In addition, the operative area can be irradiated with visible light and gooseneck lamp to raise the temperature of the flap and prevent spasms and necrosis of the blood vessels. The flap color, temperature, as well as drainage volume of the drainage ball and bottle should be observed every day to evaluate the wound exudate. Timely adoption of suitable measures such as discontinuous removal of partial suture, removal of hematoma, examination and hemostasis should be taken in case of excessive drainage volume. The suture can be removed according to the specific situation of healing if there was no obvious seepage.

## 3 Results

According to the inclusion and exclusion criteria, 33 eligible patients were enrolled in this study, including 21 males and 12 females, with an average age of 60 years (14–92 years). In terms of the specific causes of pressure injuries, there were 7 cases of chronic bed rest after cerebrovascular accident; 9 cases of paraplegia after thoracolumbar lesion; 4 cases after femur fracture; 4 cases after pelvic fracture; 1 case of long-term bed rest after arthritis; 7 patients with inability to walk due to other nervous system diseases; and 1 case with the loss of mobility after heart bypass. As for the pathological sites, 23 cases involved the sacrococcygeal region, 5 had ischiatic injuries, 3 affected the hip, and 2 got injuries in the back. According to the NPUAP staging, there were 1 case of stage III and 32 cases of stage IV. The area of pressure injury ranged from 1 cm × 1 cm to 18 cm × 11 cm. The fascial flap was used in 19 cases, the musculocutaneous flap in 5 cases, and the perforator flap in 9 cases. Postoperative flap survival was 31 cases. In 1 of the two cases without flap survival, for sacral coccygeal stage IV compressive injury, the necrotic flap was repaired with a perforator flap of the superior gluteal artery, resulting in necrosis due to flap ischemia. The patient was treated by necrotic flap removal and VSD postoperatively, with gradual healing of the wound eventually. The prognosis was good at 6 months during postoperative follow-up. In the remaining case with necrotic flap, the fascial flap was used to repair a stage IV pressure injury wound of the hip joint. The patient experienced poor wound healing after flap transfer, due to difficulty in repairing the anterior joint capsule, and hence the patient was provided with necrotic flap removal, VSD, and coverage of the wound with antibiotic-loaded bone cement to repair the wound. After the preparation of wound bed, repair using V–Y advanced flap pedicled with perforator was performed with wound healing achieved, showing good prognosis at 6 months of follow-up. Notably, 3 of the 31 survived flaps had poor healing due to ischemia, necrosis, fat liquefaction, and infection at the incisal margin of the flap. Satisfied healing was realized after the implementation of necrotic tissue resection, debridement, VSD, and intermittent suture. In one case with severe hemorrhage of flap hematoma and wound margin, the flap healed well after intermittent suture removal, hemostasis, debridement, and VSD. After 6 months of follow-up, all the 33 patients were found to have good healing, without recurrent pressure sores or infection.

## 4 Representative cases

### 4.1 Case1 bilateral V–Y propulsion flap

**Figure 1:**
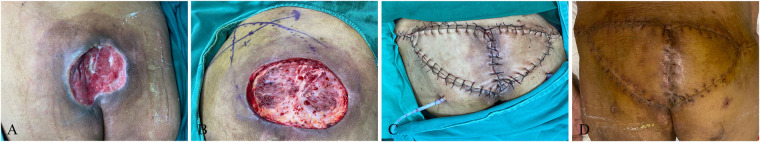
Bilateral V–Y propulsion flap. **(A)** Patient's initial wound; **(B)** flap design; **(C)** suture the flap to repair the wound; **(D)** postoperative follow-up.

A 76-year-old male patient, due to sequelae of cerebral hemorrhage, lost the ability to move his lower limbs independently and has been bedridden for a long time. The patient was observed with a stage IV pressure injury (approximately 10.0 cm × 10.0 cm in size) at the sacrococcygeal region, which had extended laterally towards the head by about 2 cm. After admission, she was given general treatments such as hypoproteinemia correction, multiple wound debridement, and VSD. With the confirmation of relatively clean wound and gradual filling of the granulation tissue, bilateral superior gluteal artery perforator flap was transferred, followed by the placement of negative pressure suction balls to fully drain the wound for blood and fluid seepage. Hypoproteinemia correction and other general treatments were continued after surgery, combined with daily irradiation using red and blue light to raise the temperature of the flap, prevent vasospasm, promote healing, with special attention paid to flap color. The patient was asked to maintain the lateral and prone position, and turn over frequently to avoid flap compression and new pressure injury. The flap healed well postoperatively, without the presence of complications such as flap necrosis or subcutaneous hematoma. Due to high tension at the junction of the flap, it triggered the formation of a liquefaction area (about 1 cm), which healed after dressing changes. The patient was observed with no contracture scar or recurrence at the wound site during postoperative follow-up (Figure 1).

### 4.2 Case 2 perforator propeller flap

**Figure 2:**
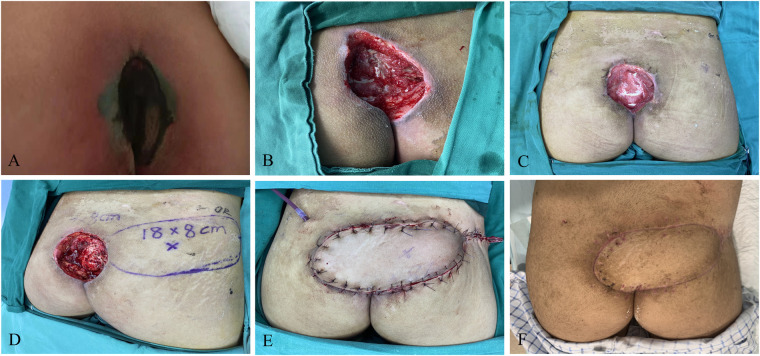
Perforator propeller flap. **(A)** Patient's initial wound; **(B)** after the first debridement; **(C)** after the VSD; **(D)** flap design; **(E)** suture the flap to repair the wound; **(F)**: postoperative follow-up.

A 14-year-old female formed a pressure injury of about 18 cm × 8 cm in size in the sacral tail (propped to the depth of 3 cm) due to prolonged bed rest resulted from weakness in both lower limbs caused by acute disseminated myelitis. After admission, active treatment was given for the primary disease, improvement of relevant etiological examination, anti-infection, as well as multiple debridement and VSD to keep the wound relatively clean. The patient's albumin was corrected to >30 g/L, and the hemoglobin was maintained to >90 g/L. Meanwhile, the patient underwent superior gluteus artery perforator screw flap transfer, with the placement of bilateral negative pressure drainage balls to fully drain the flap. The color of the flap was closely monitored, and the patient was asked to keep in the prone or left decubitus position to avoid flap compression. There were no complications such as flap necrosis, subcutaneous hematoma, and fat liquefaction, or contracture scar and recurrence during postoperative follow-up (Figure 2).

### 4.3 Case 3 ischiatic pressure sore musculocutaneous flap

**Figure 3:**
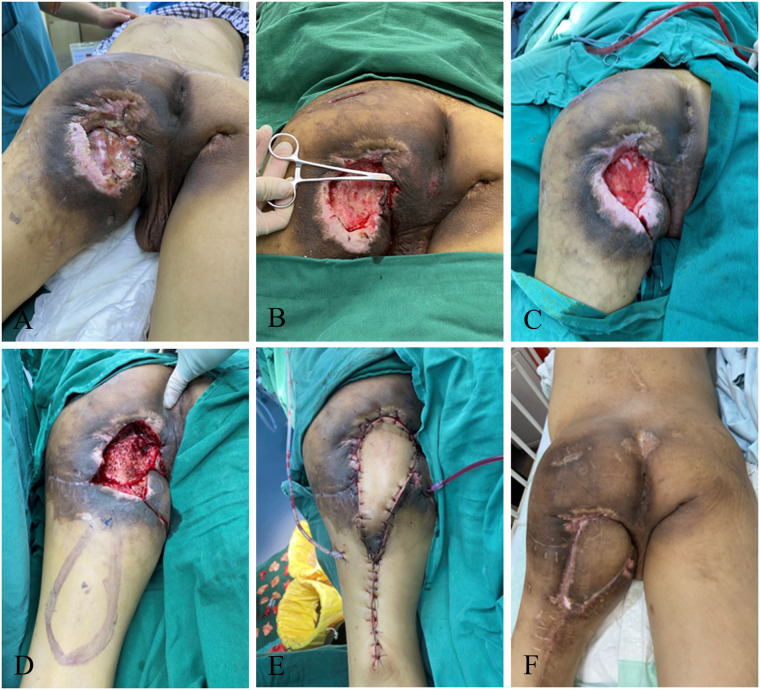
Ischiatic pressure sore musculocutaneous flap. **(A)** Patient's initial wound; **(B)** after the first debridement; **(C)** after the VSD; **(D)** flap design; **(E)** suture the flap to repair the wound; **(F)** postoperative follow-up.

A 39-year-old male with syringomyelia-induced paraplegia of the lower limb, who had been in a wheelchair for a long time, developed a pressure injury in the left ischial tubercle (about 13 cm × 10 cm) with multiple sinus canals in the buttocks. The patient received etiological examination of the wound after admission, given treatments such as anti-infection. Wound debridement and VSD were performed for the patient after full exposure of the wound. The wound was kept relatively clean, and then the long head musculocutaneous flap of the biceps femoris was transferred to the back of the thigh, with a negative pressure suction ball placed to fully remove blood and fluid from the wound. At the same time of monitoring the color of the flap, the patient was asked to maintain the lateral and prone position, and turn over frequently to avoid flap compression and new pressure injury. The patient occur bleeding of the flap after the operation, without complete healing of the incisal margin. Eventually, the patient had smooth flap healing, after intensive dressing change, and no recurrence (Figure 3).

## 5 Discussion

There is an increasing increase of pressure injuries with the aging of the population, accompanied by a deeper understanding of this disease. Currently, theories such as cell morphology, microcirculation disorder, extracellular matrix destruction, and ischemia-reperfusion are widely accepted in the exploration of the underlying mechanism of pressure injuries that are not easy to heal, among which the theory of ischemia-reperfusion stands out (13). It has been revealed that the pressure of normal skin capillaries is 16–33 mmHg, and the blood perfusion there can be blocked when tissue pressure exceeds this range, and the compression time of >2 h can lead to irreversible damage. Subsequently, local circulation disturbance may restrict the transfer of oxygen and nutrients, and failure in the clearance of inflammatory factors generated by the wound. As a result, it may further hinder the healing of wounds due to pressure injury resulted from the seepage of wound fluid, protein loss, with higher risk of infection, etc. In addition, blood reperfusion may induce damage to the vascular wall owing to a stimulated production of new reactive oxygen species and inflammatory factors, resulting in delayed healing and non-healing of the wound (14, 15). Pressure injuries usually occur in the sacrococcygeal, ischiatic tubercle, greater trochanter of the femur, lateral malleolus, and root bone. Moreover, other high-risk factors for pressure injuries may involve neurotrophic disorders, urinary incontinence, shear force, skin friction and heat, ischemia-reperfusion injury, nutritional status, Braden score ≤12, low hemoglobin level, etc. (4, 16). The elderly, being the high-risk group, are primarily featured by: (1) many underlying diseases, occurred concurrently with various diseases, such as diabetes, hypertension, hypoproteinemia, anemia, urinary and bowel incontinence, etc. The skin is thin and has poor elasticity, which is more likely to form deep pressure injury, and shows slow growth of new granulation tissue on the wound; (2) deep involvement of the pressure injury in the buttocks and ischiatic tubercles, with long-term non-healing wounds and chronic sinus formation characterized by “small opening, large base, and deep cavity”, and difficult to repair (17); and (3) high risk of systemic sepsis due to heavy local infection resulted from changes of wound cell matrix composition in the elderly, characterized by poor tolerance to surgical operations and life-threatening (18).

Traditionally, pressure injuries are usually managed through the following strategies: (1) Health education to avoid re-compression in the affected zone and prevent further wound aggravation; (2) Use of various wound dressings, such as foam dressings, hydrocolloid dressings, fibrous dressings, hydrogel dressings, etc., can contribute to mitigating compression to the affected zone and absorbing exudate; (3) Surgery: Repeated debridement, negative pressure sealing drainage, skin grafting, etc., can facilitate the removal of necrotic infected tissue and narrowing of wound surfaces. It has been recognized that flap repair is often the optimal reconstruction method for the treatment of stage III and IV pressure injury wounds. However, a fixed and single flap repair approach often fails to achieve satisfactory outcomes due to the complex nature of wounds, variations in patients' underlying conditions, and differences in the location, size, and depth of wounds. It is prone to complications such as poor wound healing, marginal necrosis, sinus tract formation, and recurrence, leading to the prolonged length of stay in the hospital and compromised patient satisfaction. Therefore, our team proposes the requirement for treatment plan formulation when treating individuals with pressure injury wounds, especially stage III and IV ones.

Currently, common flap types applied clinically include fascial flaps, perforator flaps, myocutaneous flaps, etc.

The fascial flap is usually composed of skin and subcutaneous tissue. Limberg diamond flap, Dufourmentel flap, Tai chi flap, and V–Y flap can be designed according to the characteristics of the wound, followed by wound repair through rotation, promotion, and local transfer. Limberg diamond flap is designed with four equal sides, as well as 120° and 60° triangles simultaneously. Such type of flap is subjected to relatively small tension, with the preservation of the subcutaneous vascular network as much as possible, and mild scarring after healing. However, for some wounds with large defects, Limberg diamond flap alone may fail to achieve satisfactory repair, requiring combined use of other flap types and repair methods. Compared with the Limberg diamond flap, the Dufourmentel flap, modified on the basis of the former flap type, has a wider vascular pedicle and a smaller rotation angle, which facilitates the shortening of flap-defect distance,and greatly reduces the tension of the flap and the risk of flap ischemia (19). Furthermore, Taiji flap is more suitable for sacrococcygeal pressure injuries, with advantages of less tension, more aesthetic shape. V–Y flap is a common option for wound repair due to its less damage to the donor area and satisfactory repair effect, yet accompanied by relatively larger tension after repair, and requirement of other repair methods for wounds with large defects. In our operation, in accordance with different wounds, the position and shape of the flap can be designed with a sterile marker pen, followed by the incision and separation of the flap along the marked line, and then rotation, propulsion, and translocation to repair the wound. The skin and subcutaneous tissues should be sutured discontinuously by a horizontal mattress inversion suture. Rubber strips can be placed intermittently on the closed wound for drainage. Following suture, the incisal margin should be covered with sterile dressing and the center area of the flap should be exposed to facilitate observation of flap survival. Alternatively, the opposite closure can be covered with a VSD device again for full drainage and complication prevention after the flap repair.

The musculocutaneous flap is suitable for wounds with large and deep defects frequently, with additional function of filling ineffective cavities effectively. Compared with fascial flap and perforator flap, it has rich blood supply, and is more wear-resistant, which can be useful for repairing pressure injuries at the ischiatic tubercle. However, the clinical application of this type of flap is restricted owing to bloated wound after repair, relatively large amount of blood loss during manual operation, and relatively high occurrence of complication of postoperative subcutaneous hematoma. In our surgical team, musculocutaneous flap of the biceps femoris and gluteus maximus is commonly employed to repair pressure injuries at the ischiatic tubercles. The biceps femoris, located in the posterior thigh muscle group, is designed as a V–Y musculocutaneous flap, which is then used to fully fill the tissue defect at the ischial tubercle through propulsion or local transfer. Pressure injuries at the ischial tubercle occur usually in patients who sit in a wheelchair or stay in bed for a long time and lose the ability to move their lower limbs. The use of the biceps femoris musculocutaneous flap is often the first choice, which has less impact on patients' ability to exercise. During the operation, the flap is designed according to the characteristics of the wound, followed by skin incision along the marked line, separation of the subcutaneous deep fascia tissue, exposure of the donor muscle, careful examination of the branch blood vessels in the muscle and protection of the main vessels, ligation of the surrounding small blood vessels to avoid bleeding, separation of the muscle and its blood vessels from the distal end to the near end, and finally rotation or propulsion of the flap. During operation, the blood vessels should be protected to prevent bending from affecting the survival of the later flap. After would coverage, the negative pressure drainage ball should be placed, the rubber drainage strip should be sutured layer by layer and placed intermittently, and then wound dressing with medical dressing or VSD again for sufficient drainage and complication prevention after flap transfer.

The first priorities for the use of perforator flap are preoperative identification and location of the perforator blood vessels of the required flap, as well as maintenance of the patency of blood vessels during and after surgery, so as to ensure the survival of the flap. Our team often uses the superior gluteal artery or inferior gluteal artery as the perforating branch of the flap, combined with the specific repair site, and designs into a propeller, V–Y shape for subsequent coverage of the defect through rotation and propulsion. In general, the superior gluteal artery perforator is located in the middle upper 1/3 of the line between the posterior superior iliac spine and the tip of the greater trochanter, and the inferior gluteal artery perforator located in the middle lower 1/3 of the line between the posterior iliac crest and the lateral ischiatic tubercle (20). As for the specific operations, preoperatively, Doppler ultrasound should be employed to accurately identify the perforating branches. Intraoperatively, the skin and subcutaneous tissue should be cut along the marked line, followed by careful separation and location of the blood vessels in the pedicle of the flap During flap transfer, it is critical to avoid the bending and folding of the pedicle blood vessels that may affect the survival of the flap. In addition, with the development of microsurgery, the flap-carrying perforator can also be dissociated for wound repair. The blood vessels of the flap should be anastomosed by the surgeon to the receiving area to ensure the blood supply of the flap. Following the coverage of the wound by the perforator flap, it is important to monitor the skin color and blood flow of the flap, with further placement of negative pressure drainage balls, and then layer-to-layer suture of the wound, and placement of rubber drainage strips in the intersections for full drainage to prevent the accumulation of blood underneath the flap tissue. After that, a sterile dressing should be applied to cover the closed opening of the flap and expose the center area of the flap to facilitate the observation of flap survival. Alternatively, a VSD device may be used to cover the suture to fully drain the wound and avoid postoperative complications.

In our study, through retrospective analyses, we summarized patients with pressure injuries hospitalized in our department. We concluded that for sacrococcygeal pressure injuries with small wounds, the use of a fascial flap or perforator flap of the superior gluteal and inferior gluteal artery, combined with local transfer or promotion to repair the wounds had better effects, which could greatly shorten the operation time and reduce the risk of surgery and anesthesia in the elderly. Simultaneously, according to the specific location and size of the wound, the flap can be designed into Limberg diamond flap, Dufourmentel flap, Taiji flap, V–Y flap, propeller flap, and other shapes to reduce the tension of the flap, thereby decreasing the possibility of flap and margin necrosis, and effectively preventing the split of the wound. It also reduced the risk of scarring of postoperative wound healing. For pressure injuries at the ischiatic tubercle, biceps femoris or gluteus maximus musculocutaneous flap exhibited a better effect, which could effectively fill the wound defect, so that the flap could be more wear-resistant, and the possibility of pressure injury could be lower. However, it is a relatively complicated operation to use musculocutaneous flap, accompanied by increased likelihood of blood loss, which is more suitable for patients with mild underlying diseases. Besides, it is recommended to keep the prone position after surgery to avoid flap compression. For some patients with exposed joint capsular pressure injuries, it is necessary to adequately debridement, select the appropriate method for flap repair, and reduce the exposed area of the joint capsular as much as possible to ensure the survival of the flap.

In our opinion, it is of great significance to pay attention to the whole perioperative process for the treatment of patients with pressure injuries. Given the high-risk group of the elderly with a variety of underlying diseases, corresponding primary disease should be actively treated throughout the period, combined with the control of blood pressure and blood glucose, and correction of hypoproteinemia. Furthermore, patients should be bedridden for a long time, associated with appropriate anticoagulant treatment, the use of air mattress and family member's cooperation to turn them over, thereby avoiding the aggravation of pressure injuries. Anti-infective treatment is also needed to avoid sepsis and actively prevent the occurrence of cardiovascular and cerebrovascular accidents. When performing surgery, repeated and thorough debridement are necessitated preoperatively to reduce the attachment of infected tissue as much as possible, so that the wound is attached to fresh granulation tissue to improve the survival of the flap. The key to the success of flap for repair lies in a reasonable selection of flap prosthesis according to different wound location and size. For relatively large pressure injuries, various flap relays can be selected to repair jointly. As for other surgical details, the surgeon should be familiar with the anatomical structure of the repair site. Preoperative Doppler ultrasound should be performed to localize perforator vessels to ensure the success of perforator flap repair. The operation should be conducted carefully and gently to preserve as many blood vessels as possible to maintain the blood supply of the flap and reduce the possibility of ischemic necrosis of the flap. In addition, postoperative care also occupies an important position, with special attention required to the volume of drainage of the negative pressure drainage ball. In case of excessive liquid drainage, patients should be examined in time to confirm if there are complications such as subcutaneous hematoma, and remove stitches, explore, and stop bleeding in time when necessary. Besides, the operation area can be irradiated daily with visible light, associated with the observation of the color and temperature of the flap to avoid vasospasm and other conditions that may compromise the survival of the flap. Family members of patients should be educated postoperatively, and the patient's lying position should be guided at the bedside to avoid compression and excessive tension of the flap, which may produce negative impact on flap survival, incisal margin healing, and new injury formation.

For patients who are bedridden for a long time, it is a great challenge to prevent the recurrence of pressure ulcers after repair. As believed by our team, the recurrence of pressure ulcers can be usually explained by the following reasons: (1) Given the “small opening but large base” of pressure ulcers (especially those in the ischial region), it may be impossible to implement thorough debridement, leaving a small amount of bacteria in the wound. These bacteria may further develop into a sinus tract gradually, leading to the recurrence; (2) There may be a high risk for ischial pressure ulcers to extend deep to the bone, and the presence of pseudobursa may be a risk factor for postoperative recurrence; (3) The flap may fail to fully fill the wound during flap repair, resulting in residual dead space; (4) It may be related to the poor patient compliance postoperatively, such as their inability to maintain a proper body position, excessive tension on the flap, or repeated compression of the flap; (5) Some patients with pressure ulcers are complicated with fecal and urinary incontinence. In particular, the wound is often close to the anus in patients with sacrococcygeal pressure ulcers. Improper care by family members, such as failure to timely clean feces or urine, may lead to skin maceration and trigger recurrence after repair possibly. In view of the above, it highlights the importance and necessity of full and thorough debridement in preventing recurrence. Debridement, if necessary, can be expanded to fully expose necrotic tissue. As for flap selection, it is necessary to consider the patient's general condition and choose a flap of appropriate size that can fully fill the wound, while avoiding residual space during suturing. In addition, enterostomy surgery and urinary catheterization may be reasonable choices for patients with fecal and urinary incontinence, which may reduce recurrence caused by fecal and urinary maceration. In addition, postoperative cooperation of patients and their families, coupled with strict health education, is also crucial to prevent the recurrence.

## 6 Conclusion

To sum up, for the repair of pressure injuries, flaps should be designed according to the location and size of their separate injuries. Meanwhile, emphasis should also be laid on perioperative management, as well as postoperative nursing and treatment.

## Data Availability

The raw data supporting the conclusions of this article will be made available by the authors, without undue reservation.
